# Electrolyte and Metabolic Disturbances in Ebola Patients during a Clinical Trial, Guinea, 2015

**DOI:** 10.3201/eid2212.161136

**Published:** 2016-12

**Authors:** Johan van Griensven, Elhadj Ibrahima Bah, Nyankoye Haba, Alexandre Delamou, Bienvenu Salim Camara, Kadio Jean-Jacques Olivier, Hilde De Clerck, Helena Nordenstedt, Malcolm G. Semple, Michel Van Herp, Jozefien Buyze, Maaike De Crop, Steven Van Den Broucke, Lutgarde Lynen, Anja De Weggheleire, the Ebola-Tx Consortium

**Affiliations:** Institute of Tropical Medicine, Antwerp, Belgium (J. van Griensven, J. Buyze, M. De Crop, S. Van Den Broucke, L. Lynen, A. De Weggheleire);; Donka National Hospital, Conakry, Guinea (E.I. Bah);; National Blood Transfusion Center, Conakry (N. Haba, B.S. Camara, K.J.-J. Olivier);; National Center for Training and Research in Rural Health of Maferinyah, Forecariah, Guinea (A. Delamou);; Médecins Sans Frontières, Brussels, Belgium (H. De Clerck, H. Nordenstedt, M. Van Herp);; University of Liverpool, Liverpool, United Kingdom (M.G. Semple)

**Keywords:** Ebola, EVD, electrolyte, metabolic, death, mortality, risk, prediction, model, point of care, viruses, infectious disease, clinical trial, Guinea

## Abstract

By using data from a 2015 clinical trial on Ebola convalescent-phase plasma in Guinea, we assessed the prevalence of electrolyte and metabolic abnormalities at admission and their predictive value to stratify patients into risk groups. Patients underwent testing with a point-of-care device. We used logistic regression to construct a prognostic model and summarized the predictive value with the area under the receiver operating curve. Abnormalities were common among patients, particularly hypokalemia, hypocalcemia, hyponatremia, raised creatinine, high anion gap, and anemia. Besides age and PCR cycle threshold value, renal dysfunction, low calcium levels, and low hemoglobin levels were independently associated with increased risk for death. A prognostic model using all 5 factors was highly discriminatory (area under the receiver operating curve 0.95; 95% CI 0.90–0.99) and enabled the definition of risk criteria to guide targeted care. Most patients had a very low (<5%) or very high (>80%) risk for death.

## Abstract 2

Such abnormalities were common during infection and enabled accurate stratification of the risk for death.

Electrolyte and Metabolic Disturbances in Ebola

During the 2014–2016 Ebola virus disease (EVD) outbreak in West Africa, a total of 28,646 cases were diagnosed, with a case-fatality rate of 39.4% (*1*). Several research groups have focused on new therapeutic interventions, but none has been found to be very effective (*2*–*4*). Others have emphasized the importance of good supportive care (*5*–*11*). Besides indicating appropriate oral and intravenous hydration, a body of research has also made the case for increasing access to point-of-care (POC) devices to detect metabolic and electrolyte abnormalities (*5*–*8*,*10*–*13*) because pronounced abnormalities have been observed in Ebola virus patients treated in the United States and Europe (*14*).

However, the evidence base for this recommendation in busy, resource-limited Ebola treatment units (ETUs) remains limited. If few readily treatable electrolyte disturbances would be detected with these devices, their added value could be questioned because each blood collection puts healthcare workers at risk for infection (*15*). The limited understanding of the prognostic importance of these abnormalities further obscures the evidence base on their clinical value in resource-constrained settings. Moreover, findings might differ within and between countries, and information from various settings is thus required to inform management guidelines. Despite the ≈30,000 EVD cases diagnosed worldwide over the past 40 years, the evidence base on the prevalence and prognostic value of blood abnormalities is limited to a small number of reports on a fraction of all treated Ebola patients (*4*,*16*,*17*), many of whom were being treated intensively in high-resource settings (*14*,*18*,*19*).

A better understanding of abnormal blood test results could have important clinical consequences. First, more precise information on the prevalence of abnormalities could enable rational decision-making on which testing to prioritize and which abnormalities treatment programs should prepare to appropriately manage. Identified abnormalities could lead to relevant, simple therapeutic interventions (e.g., potassium [K^+^] supplementation) that could reduce case-fatality rates. Second, identification of factors independently associated with an increased risk for death could lead to better prognostic classification of patients, identifying those in need of higher levels of care, and improve standardization when analyzing results of future clinical trials. To enable clinical decision-making, estimates of the absolute risk for death would be required for individual patients across the spectrum of independent risk factor values (*20*) and not the mere reporting of group odds or risk ratios, as has been done before (*16*,*17*). Based on the predicted absolute risk for death, individual risk stratification could be done on admission.

The Ebola-Tx trial evaluated convalescent-phase plasma as an EVD treatment in a Médecins sans Frontières (MSF) ETU in Conakry, Guinea. Trial findings have been published (*2*). MSF introduced a POC device during the trial. We used the opportunity of having access to relatively high-quality data from this clinical trial to 1) describe the prevalence of electrolyte and metabolic disturbances during admission and the association with EVD death and 2) assess the predictive value of these prognostic factors to stratify patients in risk groups.

## Methods

### Ebola-Tx Trial

The Ebola-Tx trial was conducted at the Conakry ETU supported by MSF. Trial patients were recruited during February 17–July 7, 2015. Patients of all ages, including pregnant women, were eligible, and their outcomes were compared with historical controls treated at the same center before the trial began. As per World Health Organization recommendations, patients received 2 units of 200–250 mL of Ebola convalescent-phase plasma after EVD diagnosis was confirmed by PCR (*2*). For children, a total volume of 10 mL/kg of Ebola convalescent-phase plasma was given. The level of neutralizing antibodies in the donors was unknown at the time of administration. Although the trial was found safe and feasible to organize, the efficacy of convalescent-phase plasma was not proven; the adjusted absolute risk reduction was −3% (95% CI −13% to 8%) (*2*).

### Supportive Care at the MSF ETU

Supportive care during the trial was provided by the MSF team as per MSF guidelines. As part of supportive care, MSF staff introduced a POC device (i-STAT; Abbott Point of Care, Princeton, NJ, USA) on February 25, 2015, soon after the trial began. All patients were tested at the time of EVD diagnosis by using CHEM8+ cartridges (Abbott Point of Care) to determine levels of electrolytes (K^+^, sodium [Na^+^], chloride [Cl^–^], and ionized calcium [iCa^2+^]), creatinine, and blood urea nitrogen (BUN); the anion gap; the total amount of dissolved carbon dioxide (TCO_2_); glucose levels; and hemoglobin/hematocrit values.

The MSF supportive care guidelines recommend empirical systematic prescription of antibiotics and antimalarial drugs. Symptomatic care (e.g., for pain or nausea/vomiting) was given as needed. Fluid replenishment was done with oral rehydration fluids if a patient was alert, not vomiting, and able to participate in their own care. Intravenous fluids were given to patients with insufficient oral intake, severe vomiting or diarrhea, pronounced or persistent hypotension, or clinical signs of severe dehydration (*21*).

### Study Population

A total of 102 patients were enrolled in the Ebola-Tx trial, of whom 98 received convalescent-phase plasma. A total of 87 were recruited after the introduction of the POC device.

### Data Collection

During the trial, data were entered in an electronic case report form directly from source documents, which, when filled in the high-risk zone, were scanned using mobile phones and transmitted over a secured local wireless network to a central server and automated printer. Data were collected on admission and each day after EVD confirmation, including information on symptoms and clinical signs of EVD, vital signs, and treatment received. For our study, we used all available data on the POC results on admission, cycle threshold (C_t_) values of the Ebola diagnostic PCR, baseline patient characteristics, and discharge status.

### Definitions

We used definitions based on the POC device normal values and standard definitions for abnormalities: hypokalemia, K^+^ <3.5 mmol/L; moderate hypokalemia, K^+^ 2.5–3.0 mmol/L; severe hypokalemia, K^+^ <2.5 mmol/L; hyperkalemia, K^+^ >5.0 mmol/L; hyponatremia, Na^+^ <135 mmol/L; hypernatremia, Na^+^ >146 mmol/L; hypochloremia, Cl^–^ <98; high anion gap, >20; low TCO_2_, <24 mmol/L; low glucose, <70 mg/dL; high glucose >180 mg/dL; creatinine and BUN: mild increase, 1.0–1.5 times the upper limit of normal (ULN); moderate increase, 1.5–3.0 times ULN; severe, >3.0 times ULN; increased BUN/creatinine ratio, >20; and hypocalcemia, iCa^2+^ <1.12 mmol/L. Anemia was defined per World Health Organization guidelines (age/sex adjusted) using hemoglobin values (*22*).

### Statistical Analysis

We calculated medians and interquartile ranges (IQRs) for continuous variables and summarized binary/categorical data by using frequencies and percentages. We compared groups by using Fisher exact tests for binary/categorical variables and the Wilcoxon rank-sum test for continuous variables.

The outcome status at the time of discharge from the MSF ETU was classified as alive or dead, excluding patients transferred to another ETU. We performed a risk factor analysis by using multivariate logistic regression modeling to determine independent factors associated with increased risk for death. We selected a restricted number of exposure variables on the basis of theoretical considerations and published studies. We included the following variables in the POC model: K^+^, iCa^2+^, and creatinine levels; anion gap; amount of TCO_2_; glucose levels; and hemoglobin levels. The POC+ model included the same POC variables plus age and the C_t_ of the PCR at EVD diagnosis, given that these 2 additional variables are the most universally accepted prognostic factors (*4*,*23*,*24*). To maximize power, we included the variables as continuous variables and determined the functional form by using the fracpoly command (fractional polynomial regression, which fits fractional polynomials as part of the specific regression model) in Stata version 14 (StataCorp LP, College Station, TX, USA). We reduced the model by using backward stepwise elimination until all variables had a p value <0.05. By using the logistic regression coefficients from the final model, we calculated the predicted risk for death for each patient and plotted the results in a histogram. We summarized the predictive value of the model by calculating the area under the receiver operating curve (AUROC) separately for the POC and the POC+ model.

We tabulated predicted and observed deaths across different predicted risk groups (<5.0%, 5.0%–19.9%, 20.0%–49.9%, 50.0%–79.9%, and >80.0%). We formally assessed the goodness-of-fit of the model by using the Hosmer-Lemeshow test, yielding a p value <0.05, which suggests significant discrepancy between the observed and predictive outcomes. We conducted internal validation by using cross-validation (h = 10, k = 10) (*25*). We randomly divided the data into 10 mutually exclusive subsets of the same size. We conducted a 10-fold cross-validation by repeating the analysis 10 times, each time excluding 1 of the independent datasets, and calculating the AUROC. We averaged the summary estimates of the 10 AUROCs to obtain the cross-validation AUROC. We conducted statistical analysis by using Stata version 14.

## Results

Of the 87 study patients enrolled in the trial when the POC device was in use, POC results were available for 85 (Table 1). The median age was 30 years (IQR 20–40 years); 48 (56%) patients were female. The median C_t_ value in the diagnostic PCR was 27 (IQR 18–36).

**Table 1:** Baseline characteristics of 85 Ebola patients recruited for the Ebola-Tx trial, Conakry, Guinea, 2015*

Characteristic	Value
Age, median y (IQR)	30 (20–40)
<15	10 (11.8)
Sex	
M	37 (43.5)
F	48 (56.5)
C_t_ value on diagnostic Ebola PCR, n = 84	
No. cycles, median (range)	26.8 (17.8–35.8)
<25	23 (27.4)
25.0–29.9	40 (47.6)
≥30	21 (25.0)
Duration of symptoms, median d (IQR), n = 74	4 (3–5)
Coexisting chronic medical condition	15 (17.6)
Infectious†	8 (9.4)
Noninfectious‡	7 (8.2)
Selected symptoms on admission	
Nausea and vomiting	43 (50.6)
Diarrhea	29 (34.1)

*Values are no. (%) patients except as indicated. C_t_, cycle threshold; IQR, interquartile range. †For example, tuberculosis, HIV, or malaria. ‡For example, diabetes mellitus, arterial hypertension, chronic cardiac, pulmonary disease, or renal disease.

Hyponatremia (77.6%) was very common, but hypernatremia was only rarely documented (Table 2). Although hypokalemia (33%) was common, severe hypokalemia (K^+^ <2.5 mmol/L) and hyperkalemia were rare. Hypocalcemia (64%) was frequently observed. Renal dysfunction (45% with increased creatinine levels), an increased anion gap (28%), and decreased TCO_2_ (71%) all were frequently documented. Anemia was observed in 27% of patients. 

**Table 2:** Metabolic and electrolyte disturbances in 85 Ebola patients recruited for the Ebola-Tx trial and association with increased risk for death, Conakry, Guinea, 2015*

Analyte	Value			p value
	Total	Deceased	Survived	
Total†	85	27	51	
K^+^, mmol/L median (IQR)	3.7 (3.2–4.2)	3.9 (3–2-4.7)	3.7 (3.2–4.0)	0.21
High	4 (4.7)	3 (11.1)	0 (0)	
Normal	53 (62.3)	16 (59.3)	33 (64.7)	
Mild decrease	12 (14.1)	4 (14.8)	7 (13.7)	0.19
Moderate decrease	14 (16.5)	4 (14.8)	9 (17.6)	
Severe decrease	2 (2.3)	0 (0)	2 (3.9)	
Na^+^, mmol/L median (range)	135 (132–137)	132 (129–138)	136 (133–137)	0.055
Low	66 (77.6)	20 (74.1)	39 (76.5)	
Normal	18 (21.2)	6 (22.2)	12 (23.5)	0.52
High	1 (1.2)	1 (3.7)	0 (0)	
Cl^–^, mmol/L median (IQR)	99 (95–103)	101 (97–105)	99 (96–102)	0.41
Low	34 (40.0)	10 (37.0)	18 (35.3)	1.0
iCa^2+^, mmol/L median (IQR)	4.3 (4.0–4.6)	3.9 (3.5–4.2)	4.4 (4.2–4.7)	<0.01
Low	54 (63.5)	24 (88.9)	26 (51.0)	<0.01
TCO_2_, mmol/L median (IQR	21 (18–24)	17 (16–22)	21 (19–25)	<0.01
Low	60 (70.6)	23 (85.2)	37 (72.5)	0.27
Anion gap, mmol/L median (IQR)	19 (17–21)	19 (18–21)	18 (17–20)	0.26
High	24 (28.4)	11 (40.7)	10 (19.6)	0.061
Creatinine, mmol/L median (IQR), n = 84	106 (75–332)	442 (148–654)	91 (63–116)	<0.01
Normal	47 (55.9)	5 (18.5)	38 (74.5)	
1–3x ULN	16 (19.0)	5 (18.5)	9 (17.6)	<0.01
>3x ULN	21 (25.0)	17 (63.0)	4 (7.8)	
BUN, mmol/L median (IQR), n = 84	16 (9–39)	50 (17–69)	11 (7–21)	<0.01
Normal	53 (63.1)	9 (33.3)	38 (76.0)	
1–3x ULN	26 (30.9)	13 (48.1)	12 (24.0)	<0.01
>3x ULN	5 (6.0)	5 (18.5)	0 (0.0)	
BUN/creatinine ratio, median (IQR), n = 83	10.3 (7.4–13.9)	8.8 (6.8–13.9)	11.2 (7.7–14.0)	0.30
<10	36 (43.4)	15 (55.6)	18 (36.0)	
10–20	39 (47.0)	10 (37.0)	26 (52.0)	0.27
>20	8 (9.6)	2 (7.4)	6 (12.0)	
Glucose, mmol/L median (IQR)	121 (104–148)	112 (88–146)	122 (106–147)	0.24
Low	5 (5.9)	4 (14.8)	1 (2.0)	
Normal	72 (84.7)	19 (70.4)	47 (92.2)	0.022
High	8 (9.4)	4 (14.8)	3 (5.9)	
Hemoglobin, g/dL median (IQR)	14.6 (11.9–16)	13.6 (10.5–17)	14.3 (11.9–15.6)	0.90
Anemia‡	23 (27.1)	9 (33.3)	14 (27.4)	0.61

*Values are no. (%) patients except as indicated. BUN, blood urea nitrogen; C_t_, cycle threshold; iCa^2+^, ionized (free) calcium; IQR, interquartile range; K^+^, potassium; Na^+^, sodium; Cl^–^, chloride; TCO_2_, total dissolved carbon dioxide; ULN, upper limit of normal value. †Denominator for deceased and survived patients combined is 78, excluding 7 patients who were transferred to another Ebola treatment unit, where they received favipiravir.  ‡Definition of anemia per World Health Organization guideline (age/sex adjusted; hemoglobin levels used).

**Figure 1:**
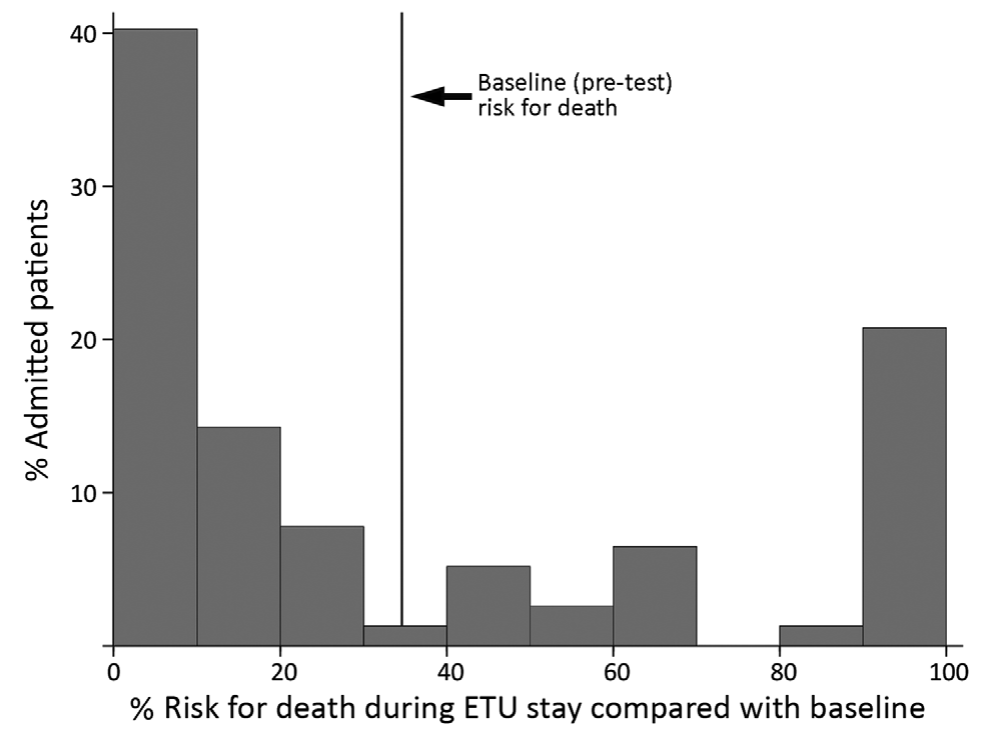
Histogram displaying the distribution of the risk for death for Ebola patients recruited for the Ebola-Tx trial, Conakry, Guinea, 2015, according to a 5-variable point-of-care (POC+) prognostic prediction model. POC+ model includes 3 POC measurements (blood creatinine, calcium, and hemoglobin) plus the cycle threshold value of the diagnostic Ebola PCR result and the age of the patient. ETU, Ebola treatment unit.

**Figure 2:**
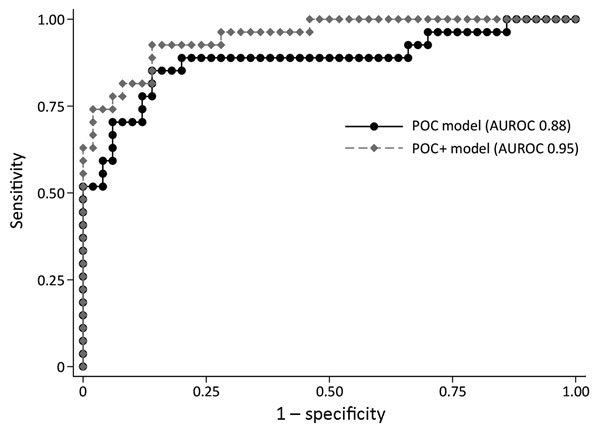
Receiving operating curve summarizing the performance of 3-variable point-of-care (POC) and 5-variable POC+ prognostic prediction models for Ebola patients recruited for the Ebola-Tx trial, Conakry, Guinea, 2015. POC model includes blood creatinine, hemoglobin, and calcium levels. POC+ model includes the same 3 POC measurements plus the cycle threshold value of the diagnostic Ebola PCR result and the age of the patient. AUROC, area under the receiver operating curve; POC, point-of-care.

In the multivariate logistic regression modeling, decreased iCa^+^ and hemoglobin levels and increased creatinine levels were associated with increased risk for death (POC model). In the POC+ model, age and the diagnostic PCR C_t_ value were additionally selected (Table 3). A prediction model based on these 5 factors provided estimates of the risk for death ranging from 0.016% to 99.99%, compared with a baseline (or pretest) risk for death of 34.6% (27/78 patients). The largest risk groups were patients at either very low or very high risk, with 40% of patients having a risk for death <10% and 22% having a risk >80% (Figure 1). This POC+ model had an AUROC of 0.95 (95% CI 0.90–0.99), compared with 0.88 (95% CI 0.78–0.97) for the model that only included creatinine, calcium, and hemoglobin levels (POC model) (Figure 2). AUROC cross-validation values were 0.92 (95% CI 0.85–0.99) for the POC+ model and 0.85 (95% CI 0.75–0.96) for the POC model. The Hosmer-Lemeshow test yielded a p value of 0.98 for the POC+ model and 0.34 for the term POC model. The predicted and observed risk for death was stratified into 5 risk groups (Table 4); most patients were in the <5% or >80% risk group categories.

**Table 3:** Factors associated with increased risk for death among 78 Ebola patients recruited for the Ebola-Tx trial, Conakry, Guinea, 2015*

Blood test result†	POC model		POC+ model,‡ aOR (95% CI)
	Crude OR (95% CI)	aOR (95% CI)	
K^+^, per unit increase	1.7 (0.9–3.3)	–	–
iCa^2+^, per 0.1-unit increase)	0.70 (0.59–0.84)	0.78 (0.63–0.96)	0.73 (0.57–0.95)
Glucose, per 50-unit increase)	0.87 (0.50–1.52)	–	–
Creatinine, per 100-unit increase)	2.1 (1.5–3.0)	2.1 (1.3–3.3)	2.3 (1.1–4.6)
TCO_2_, per 5-unit increase)	0.38 (0.20–0.74)	–	–
Anion gap, per 5-unit increase)	1.5 (0.6–3.7)	–	–
Hemoglobin, per 1-unit increase)	1.0 (0.9–1.2)	0.79 (0.63–0.99)	0.67 (0.47–0.93)
C_t_ value, per 1-unit increase)	0.80 (0.70–0.93)		0.73 (0.56–0.94)
Age, per 10-y increase)	1.8 (1.3–2.6)		2.7 (1.2–6.4)

*aOR, adjusted odds ratio; C_t_, cycle threshold; iCa^2+^, ionized (free) calcium; K^+^, potassium; OR, odds ratio; POC, point-of-care; TCO_2_, total dissolved carbon dioxide. †No evidence of nonlinearity of the continuous variables except for glucose, which was transformed to the power of –2. ‡POC+ model includes 3 POC measurements (blood creatinine, calcium, and hemoglobin) plus the cycle threshold value of the diagnostic Ebola PCR result and the age of the patient.

**Table 4:** Predicted and observed deaths across risk categories among 77 Ebola patients recruited for the Ebola-Tx trial, according to the POC+ model, Conakry, Guinea, 2015*†

Death risk category (predicted), %	Total no. patients (column %)	No. deaths observed (row %)	No. deaths predicted (row %)
0–4.9	28 (36.4)	1 (3.6)	0.4 (1.5)
5.0–19.9	14 (18.2)	1 (7.1)	1.7 (12.3)
20.0–49.9	11 (14.3)	4 (36.4)	3.7 (34.0)
50.0–79.9	7 (9.1)	4 (57.1)	4.4 (63.5%)
80.0–100	17 (22.1)	17 (100.0)	16.7 (98.1)

*One recruited patient was excluded because of a missing C_t_ value. C_t_, cycle threshold; POC, point-of-care. †POC+ model includes 3 POC measurements (blood creatinine, calcium, and hemoglobin) plus the cycle threshold value of the diagnostic Ebola PCR result and the age of the patient.

## Discussion

We documented a high prevalence of multiple electrolyte and metabolic abnormalities in Ebola patients at the time of admission during a clinical trial of convalescent-phase plasma in Guinea. Increased creatinine and decreased calcium and hemoglobin levels during admission were independent risk factors for death. The POC model including these factors and the POC+ model additionally including 2 key risk factors (C_t_ value of the diagnostic Ebola PCR and the age of the patient) both performed well in predicting individual patient outcome. The predicted risk for death ranged from <0.02% to 99.9%, and most patients were found to have either a very low risk (<5%) or a very high risk (>80%) for death.

The frequent observance of electrolyte disturbances is in line with the few available studies from Africa (*17*,*26*). The high prevalence of hypokalemia is of particular interest, although it was rarely severe at the time of admission. Nevertheless, hypokalemia can potentially be fatal and is amenable to relatively simple interventions. A deficiency in iCa^+^ was common, a finding also frequently observed in sepsis patients (*27*,*28*). In the case of EVD, renal dysfunction and pancreatitis also could contribute. The high anion gap and low TCO_2_ are likely explained by lactic acidosis. Renal failure was also common in our study. The exact cause of renal dysfunction in EVD patients remains undefined, but both prerenal and renal causes probably are involved.

The POC+ model, a 5-measure prognostic tool, enabled the stratification of patients into different risk groups at baseline, with risk for death ranging from <5% to >80%. This tool could enable the refining of clinical care pathways and rational use of scarce human resources. Absolute risks for death could easily be calculated via a spreadsheet or smartphone application. Alternatively, scoring systems could be developed for bedside use (*29*,*30*). 

However, several issues need to be addressed before such prognostic tools are taken forward. First, we did not include other potentially relevant measures that can be tested with POC devices, such as liver function tests, coagulation abnormalities, or markers of inflammation. Although our system performed fairly well with only a few markers, evaluation of the full panel of tests could possibly lead to further improvement. Second, the sample size in our study was limited, and all but 2 of the patients received convalescent-phase plasma. Although transfusion of 500 mL of plasma probably will not strongly influence the prognostic associations, larger studies in different study populations are required to assess the generalizability of our findings. Third, corrective measures for some abnormalities (e.g., hypoglycemia or hypokalemia) could have obscured associations with survival. Moreover, because this was an exploratory study, our findings need to be validated in other and larger datasets. Finally, it remains to be seen whether (and if so how much) our prognostic tool would perform substantially better than clinical judgement alone or a prognostic tool relying on clinical signs/symptoms combined with age and the PCR C_t_ value. Nevertheless, a prognostic clinical tool using laboratory measures will have the advantage of being more objective, less variable between clinicians, and less dependent on specific clinical skills.

In contrast to fixed variables such as age and the PCR C_t_ value that are only useful as prognostic markers, the detection of hypocalcemia, anemia, and renal dysfunction is amenable to interventions. If these conditions are causally associated with an increased risk for death, their detection could contribute to reducing case-fatality rates. If they are only a proxy for another causal factor, correction of these abnormalities might have minimal effects. For instance, surprisingly limited evidence exists that correction of hypocalcemia in intensive care patients improves survival (*27*), and some evidence suggests that it could even be detrimental (*28*). Similarly, whether blood transfusion in cases of anemia can improve EVD survival (and at what threshold hemoglobin value) requires further study. Anemia has been found to be associated with an increased risk for death in other inflammatory conditions such as sepsis (*31*). However, subsequent studies demonstrated that a liberal blood transfusion strategy could have a negative impact on survival in intensive care patients (*32*).

The association of renal dysfunction with an increased risk for death has been demonstrated before (*4*,*17*,*26*,*33*). However, the added clinical value of being able to diagnose renal dysfunction, beyond its prognostic value, in the typical ETU in countries of Africa remains unclear. It could enable staff to more carefully assess the fluid status and thus avoid over- and under-hydration. However, renal dysfunction could be prerenal (requiring aggressive fluid administration) or renal (requiring more cautious fluid administration) in origin. Carefully assessing fluid status in busy ETUs with brief patient contact remains challenging, and to what extent the ratio of BUN to creatinine is accurate in EVD patients is unclear (*17*). Whether biochemical diagnosis (and management) of kidney dysfunction substantially improves patient outcomes compared with monitoring urine output and clinically assessing fluid status also remains unknown. It would be more useful to monitor for renal dysfunction if compensatory measures such as dialysis could be put in place (*34*). Clinical trials would be required to assess this further, looking at case-fatality rate reduction, risk to healthcare staff, and opportunity costs (*9*). As blood transfusion requires blood group testing and, ideally, bedside cross-matching, risk-benefit assessments for this intervention would also be useful to inform treatment guidelines.

We acknowledge that underestimation of the prevalence of abnormalities is probable because as measurements were not systematically performed during the entire stay in the ETU. Still, our findings show that a POC device is practical to use in the ETU and could be useful in stratifying patients into risk groups at baseline. Information on the extent POC results were used by the clinicians would also have strengthened the study.

In conclusion, in the challenging environment of an ETU, staff wearing full protective equipment were able to use a POC device that frequently detected metabolic and electrolyte abnormalities among EVD patients at admission. Besides age and diagnostic PCR C_t_ value, renal dysfunction, low calcium levels, and low hemoglobin levels were independently associated with an increased risk for death. A clinical prognostic model using these 5 factors had a high discriminatory potential, with most patients having either very low (<5%) or very high (>80%) risk for death. To what extent interventions aiming at correcting the observed abnormalities can reduce case-fatality rates remains to be assessed. Moreover, risk-benefit assessments that consider the risks to healthcare workers are required to inform treatment guidelines.

## References

[1] World Health Organization. Ebola situation report—30 March 2016 [cited 2016 Jun 1]. http://apps.who.int/ebola/current-situation/ebola-situation-report-30-march-2016

[2] van Griensven J, Edwards T, de Lamballerie X, Semple MG, Gallian P, Baize S, et al.; Ebola-Tx Consortium. Evaluation of convalescent plasma for Ebola virus disease in Guinea. N Engl J Med. 2016;374:33–42. 10.1056/NEJMoa1511812 26735992 PMC5856332

[3] Dunning J, Sahr F, Rojek A, Gannon F, Carson G, Idriss B, et al.; RAPIDE-TKM trial team. Experimental treatment of Ebola virus disease with TKM-130803: a single-arm phase 2 clinical trial. PLoS Med. 2016;13:e1001997. 10.1371/journal.pmed.1001997 27093560 PMC4836798

[4] Sissoko D, Laouenan C, Folkesson E, M’Lebing AB, Beavogui AH, Baize S, et al.; JIKI Study Group. Experimental treatment with favipiravir for Ebola Virus Disease (the JIKI trial): a historically controlled, single-arm proof-of-concept trial in Guinea. PLoS Med. 2016;13:e1001967. 10.1371/journal.pmed.1001967 26930627 PMC4773183

[5] Fletcher TE, Fowler RA, Beeching NJ. Understanding organ dysfunction in Ebola virus disease. Intensive Care Med. 2014;40:1936–9. 10.1007/s00134-014-3515-1 25366120

[6] Fowler RA, Fletcher T, Fischer WA II, Lamontagne F, Jacob S, Brett-Major D, et al. Caring for critically ill patients with ebola virus disease. Perspectives from West Africa. Am J Respir Crit Care Med. 2014;190:733–7. 10.1164/rccm.201408-1514CP 25166884

[7] Lamontagne F, Clément C, Fletcher T, Jacob ST, Fischer WA II, Fowler RA. Doing today’s work superbly well—treating Ebola with current tools. N Engl J Med. 2014;371:1565–6. 10.1056/NEJMp1411310 25251518

[8] Wong KK, Perdue CL, Malia J, Kenney JL, Peng S, Gwathney JK, et al.; Monrovia Medical Unit. Supportive care of the first 2 Ebola virus disease patients at the Monrovia Medical Unit. Clin Infect Dis. 2015;61:e47–51. 10.1093/cid/civ420 26021993

[9] West TE, von Saint André-von Arnim A. Clinical presentation and management of severe Ebola virus disease. Ann Am Thorac Soc. 2014;11:1341–50. 10.1513/AnnalsATS.201410-481PS 25369317

[10] Chertow DS, Kleine C, Edwards JK, Scaini R, Giuliani R, Sprecher A. Ebola virus disease in West Africa—clinical manifestations and management. N Engl J Med. 2014;371:2054–7. 10.1056/NEJMp1413084 25372854

[11] Hunt L, Lee JS. Empiric intravenous fluid and electrolyte therapy in patients with Ebola virus disease. Trop Doct. 2016;46:148–50. 10.1177/0049475516644883 27106251

[12] Palich R, Gala JL, Petitjean F, Shepherd S, Peyrouset O, Abdoul BM, et al.; ALIMA N’zérékoré Ebola Treatment Center medical group. A 6-year-old child with severe Ebola virus disease: laboratory-guided clinical care in an Ebola treatment center in Guinea. PLoS Negl Trop Dis. 2016;10:e0004393. 10.1371/journal.pntd.0004393 27011342 PMC4806864

[13] Clay KA, Johnston AM, Moore A, O’Shea MK. Targeted electrolyte replacement in patients with Ebola virus disease. Clin Infect Dis. 2015;61:1030–1. 10.1093/cid/civ435 26056238

[14] Uyeki TM, Mehta AK, Davey RT Jr, Liddell AM, Wolf T, Vetter P, et al.; Working Group of the U.S.–European Clinical Network on Clinical Management of Ebola Virus Disease Patients in the U.S. and Europe. Clinical management of Ebola virus disease in the United States and Europe. N Engl J Med. 2016;374:636–46. 10.1056/NEJMoa1504874 26886522 PMC4972324

[15] Kok J, Sintchenko V, Dwyer DE, Chen SC. Editorial: Laboratory preparedness for Ebolavirus disease. [editorial]. Pathology. 2015;47:397–9. 10.1097/PAT.0000000000000290 26126052

[16] Hunt L, Knott V. Serious and common sequelae after Ebola virus infection. Lancet Infect Dis. 2016;16:270–1. 10.1016/S1473-3099(15)00546-0 26725445

[17] Rollin PE, Bausch DG, Sanchez A. Blood chemistry measurements and D-Dimer levels associated with fatal and nonfatal outcomes in humans infected with Sudan Ebola virus. J Infect Dis. 2007;196(Suppl 2):S364–71. 10.1086/520613 17940972

[18] Lyon GM, Mehta AK, Varkey JB, Brantly K, Plyler L, McElroy AK, et al.; Emory Serious Communicable Diseases Unit. Clinical care of two patients with Ebola virus disease in the United States. N Engl J Med. 2014;371:2402–9. 10.1056/NEJMoa1409838 25390460

[19] Kreuels B, Wichmann D, Emmerich P, Schmidt-Chanasit J, de Heer G, Kluge S, et al. A case of severe Ebola virus infection complicated by gram-negative septicemia. N Engl J Med. 2014;371:2394–401. 10.1056/NEJMoa1411677 25337633

[20] Steyerberg EW, Moons KG, van der Windt DA, Hayden JA, Perel P, Schroter S, et al.; PROGRESS Group. Prognosis Research Strategy (PROGRESS) 3: prognostic model research. PLoS Med. 2013;10:e1001381. 10.1371/journal.pmed.1001381 23393430 PMC3564751

[21] Sterck A. Filovirus haemorrhagic fever guideline [cited 2016 Jun 1]. http://www.medbox.org/ebola-guidelines/filovirus-haemorrhagic-fever-guideline/preview

[22] World Health Organization. Haemoglobin concentrations for the diagnosis of anaemia and assessment of severity. Geneva: The Organization; 2011.

[23] Bah EI, Lamah MC, Fletcher T, Jacob ST, Brett-Major DM, Sall AA, et al. Clinical presentation of patients with Ebola virus disease in Conakry, Guinea. N Engl J Med. 2015;372:40–7. 10.1056/NEJMoa1411249 25372658

[24] Faye O, Andronico A, Faye O, Salje H, Boëlle PY, Magassouba N, et al. Use of viremia to evaluate the baseline case fatality ratio of Ebola virus disease and inform treatment studies: a retrospective cohort study. PLoS Med. 2015;12:e1001908. 10.1371/journal.pmed.1001908 26625118 PMC4666644

[25] Harrell FE Jr, Lee KL, Mark DB. Multivariable prognostic models: issues in developing models, evaluating assumptions and adequacy, and measuring and reducing errors. Stat Med. 1996;15:361–87. 10.1002/(SICI)1097-0258(19960229)15:4<361::AID-SIM168>3.0.CO;2-4 8668867

[26] Hunt L, Gupta-Wright A, Simms V, Tamba F, Knott V, Tamba K, et al. Clinical presentation, biochemical, and haematological parameters and their association with outcome in patients with Ebola virus disease: an observational cohort study. Lancet Infect Dis. 2015;15:1292–9. 10.1016/S1473-3099(15)00144-9 26271406

[27] Forsythe RM, Wessel CB, Billiar TR, Angus DC, Rosengart MR. Parenteral calcium for intensive care unit patients. Cochrane Database Syst Rev. 2008;4:CD006163. 10.1002/14651858.CD006163.pub2 18843706 PMC13716406

[28] Collage RD, Howell GM, Zhang X, Stripay JL, Lee JS, Angus DC, et al. Calcium supplementation during sepsis exacerbates organ failure and mortality via calcium/calmodulin-dependent protein kinase kinase signaling. Crit Care Med. 2013;41:e352–60. 10.1097/CCM.0b013e31828cf436 23887235 PMC3812408

[29] van Griensven J, Phirum L, Thai S, Buyze J, Lynen L. A clinical prediction score for targeted creatinine testing before initiating tenofovir-based antiretroviral treatment in Cambodia. J Acquir Immune Defic Syndr. 2014;65:e150–2. 10.1097/QAI.0000000000000022 24121759

[30] van Griensven J, Phan V, Thai S, Koole O, Lynen L. Simplified clinical prediction scores to target viral load testing in adults with suspected first line treatment failure in Phnom Penh, Cambodia. PLoS One. 2014;9:e87879. 10.1371/journal.pone.0087879 24504463 PMC3913697

[31] Hébert PC, Wells G, Tweeddale M, Martin C, Marshall J, Pham B, et al. Does transfusion practice affect mortality in critically ill patients? Transfusion Requirements in Critical Care (TRICC) Investigators and the Canadian Critical Care Trials Group. Am J Respir Crit Care Med. 1997;155:1618–23. 10.1164/ajrccm.155.5.9154866 9154866

[32] Hébert PC, Wells G, Blajchman MA, Marshall J, Martin C, Pagliarello G, et al. A multicenter, randomized, controlled clinical trial of transfusion requirements in critical care. Transfusion Requirements in Critical Care Investigators, Canadian Critical Care Trials Group. N Engl J Med. 1999;340:409–17. 10.1056/NEJM199902113400601 9971864

[33] Schieffelin JS, Shaffer JG, Goba A, Gbakie M, Gire SK, Colubri A, et al.; KGH Lassa Fever Program; Viral Hemorrhagic Fever Consortium; WHO Clinical Response Team. Clinical illness and outcomes in patients with Ebola in Sierra Leone. N Engl J Med. 2014;371:2092–100. 10.1056/NEJMoa1411680 25353969 PMC4318555

[34] Faubel S, Franch H, Vijayan A, Barron MA, Heung M, Liu KD, et al. Preparing for renal replacement therapy in patients with the Ebola virus disease. Blood Purif. 2014;38:276–85. 10.1159/000371530 25675963

